# Shape as a Key to Taxonomy: Morphometric Analysis of *Tetropium* Species (Coleoptera: Cerambycidae)

**DOI:** 10.3390/insects16040386

**Published:** 2025-04-04

**Authors:** Allan H. Smith-Pardo, Steven W. Lingafelter, David Laroze, Alejandro Piñeiro-Gonzalez, Hugo A. Benítez

**Affiliations:** 1 United States Department of Agriculture (USDA)-Animal and Plant Health Inspection Service (APHIS)-Plant Protection and Quarantine (PPQ)-Science and Technology (S&T), Pest Identification Technology Laboratory (PITL), Sacramento, CA 95814, USA; 2 United States Department of Agriculture-Animal and Plant Health Inspection Service-Plant Protection and Quarantine, Douglas, AZ 85607, USA; steven.w.lingafelter@usda.gov; 3 Instituto de Alta Investigación, Universidad de Tarapacá, Arica 1000000, Chile; dlarozen@uta.cl; 4 Vicerrectoría de Investigación y Postgrado, Universidad de La Serena, La Serena 1700000, Chile; andresale96@gmail.com; 5 Instituto One Health, Facultad de Ciencias de la Vida, Universidad Andrés Bello, República 440, Santiago 8370134, Chile; 6 Laboratorio de Ecología y Morfometría Evolutiva, Centro de Investigación de Estudios Avanzados del Maule, Universidad Católica del Maule, Talca 3466706, Chile; 7 Research Ring in Pest Insects and Climate Change (PIC2), Santiago 8320000, Chile; 8 Millennium Institute Biodiversity of Antarctic and Sub-Antarctic Ecosystems (BASE), Santiago 7800003, Chile

**Keywords:** spruce longhorned beetle, coniferous pest, spondylidinae, geometric morphometrics, shape analysis

## Abstract

The study of shape by the use of geometric morphometrics has been an important tool for addressing taxonomic challenges in complex groups like the genus *Tetropium* Kirby, 1837 (Coleoptera, Cerambycidae). This insect genus includes 28 species, 8 of which are found in North America, with the invasive *T. fuscum* (Fabricius) posing a significant quarantine risk as a pest of coniferous trees. The present study evaluated the use of geometric morphometrics to analyze the pronotum shape in females of nine species of the genus, showing the effectiveness of this tool in distinguishing between species. Even if some overlaps were found between some species, this research highlights the potential of GM in developing pest monitoring, quarantine managements, and integrated pest management programs. Our findings suggest that the use of a comprehensive database of landmarks, encompassing broader geographic and ecological diversity, could further improve species identification at ports of entry and facilitate trade.

## Simple Summary

Geometric morphometrics is an effective tool for resolving taxonomic challenges in the genus *Tetropium* (Coleoptera, Cerambycidae), which includes invasive pest species such as *Tetropium fuscum*. This study analyzed the pronotum shape in nine species, demonstrating the power of the method to differentiate them, despite some degree of overlap. The findings emphasize the potential of geometric morphometrics for pest monitoring, quarantine management, and species identification. Establishing a comprehensive database of landmarks that encompasses broader geographic and ecological diversity could further enhance the accuracy of species identification at ports of entry, facilitating international trade and strengthening biosecurity measures.

## 1. Introduction

*Tetropium* Kirby, 1837 (Coleoptera, Cerambycidae) contains 28 species that are mostly found in Europe, Asia, Central America, and North America, of which 8 species, including the invasive *T. fuscum* (Fabricius), are present in Canada and the United States [1,2]. The genus is distinctive among members of Spondylidinae in having completely divided eyes with separate upper and lower lobes. A key to most species of the United States and Canada (except for *T. auripilus* Bates) was provided in Smith & Hurley [1] (2017). The key divides the genus *Tetropium* into two groups based on the presence or absence of raised asperities on the pronotum. Further division of species is based on the punctation pattern and the shape and development of the trochanter, although both features are variable and often challenging to interpret. Among the eight species of *Tetropium* that are found in North America, seven are native and not considered quarantine-significant: *Tetropium abietis* Fall, *T. cinnamopterum* Kirby, *T. parallelum* Casey, *T. parvulum* Casey, *T. schwarzianum* Casey, *T. auripilis* Bates, and *T. velutinum* LeConte. However, *Tetropium fuscum*, introduced into eastern Canada from Europe [1], is a quarantine concern due to its non-native status and its development in conifers. Additionally, two European species, *T. castaneum* (Linnaeus) and *T. gabrieli* Weise, are not established in North America but are of quarantine significance because of their frequent interception at ports of entry and their potential to attack a wide range of coniferous trees.

The grouping of these species has been mostly focused on the interaction with forestry and agroecological systems, where an accurate identification of these beetles is critical for understanding their biology, ecology, and potential economic impacts. While traditional morphometrics methods have long been used for their identification, taxon-specific methodologies and expertise are essential for distinguishing species which have cryptic morphological characteristics [3]. In line with this need, numerous studies have utilized both molecular and morphological approaches to explore species identification [4,5,6]. Additionally, new genomic approaches have been employed to address taxonomic challenges and their evolutionary implications [7]. In the same context of developing methodologies to produce the best descriptions, geometric morphometrics (GM) enables the analysis of shape, which is defined as the geometric properties that remain after removing the effects of scale, rotation, and translation [8]. Consequently, GM provides enhanced biological insights by offering a graphical tool to visualize and quantify morphological variation across diverse ecological and evolutionary contexts [9,10]. The literature on the use of GM with beetles is limited, even though Coleoptera is the most diverse order of organisms on Earth. In addition, most of the already published papers focus primarily on agricultural species in the Chrysomelidae [11,12,13] and, to some extent, on less agriculturally related families such as Cantharidae [14], Lampyridae [15], and Lucanidae [16].

Very few publications are available on the use of geometric morphometrics for beetles of the family Cerambycidae [17,18,19,20,21]. Earlier works using GM by Rossa et al. [19,20] focused on identifying sibling species or species in species complexes. Rossa et al. [19] evaluated the use of GM in the identification of two sibling species: *Leiopus nebulosus* (Linnaeus) and *Leiopus linnei* Wallin, Nylander & Kvamme, 2009 which are also very similar morphologically, making their identification difficult and possible only with the assistance of experts in the group; in their work, they found that the identification of *L. nebulosus* and *L. linnei* based on hind wings measurements was possible, leading to the correct identification of *L. nebulosus* (95.56%) and of *L. linnei* (97.39%), and that this method also facilitated the reliable identification of both species by less experienced entomologists. On the other hand, Rossa et al. [20] quantified the morphological variation among European and Asiatic populations of *Leptura annularis* Fabricius, 1801 and its closely related species *Leptura mimica* Bates, endemic to Japan and Sakhalin islands, both of which are collectively called “*Leptura annularis* complex”. The authors found that when using hindwing landmarks, the level of morphological divergence between most continental European and Asiatic populations was relatively small and proportional to the geographic distance between them. Still, there was a distinct morphotype in Sakhalin Island and Japan, and the authors concluded that the data confirmed the morphological divergence of the endemic *L. mimica* species and proved that the geometric morphometric method is robust and applicable for studying morphological variation in beetles.

Subsequent works by Ospina-Garcés et al. [18] on saproxylic cerambycid beetles (those that depend on deadwood for larval development) and the environmental pressure they cause (due to the choice of host plants and/or wood in a particular stage of degradation) act as an environmental pressure on the head morphology of larvae and its plasticity, depending on the number of woody plant species that are used for larval development in each insect species. The authors found that generalist species using host plants showed significant head shape and size variation. In contrast, the time of emergence and season did not appear to affect the head shape, although the season was a determining factor of abundance and possibly head size variation.

Okutaner and Sariyaka [21] evaluated the sexual dimorphism of the pronotum in the species *Dorcadion micans* J. Thomson, 1867 (Coleoptera: Cerambycidae), a Turkish endemic species, using GM and found statistically significant sexual size and shape dimorphism in the pronotum of the species, with males having a smaller pronotum size than females and a pronotum shape that was longer and sharper; they also found that the overall size had a negligible influence on the differentiation in pronotum shape between sexes.

Given the challenges of identifying closely related species regarding their morphology, the present research will use geometric morphometrics as a powerful tool for addressing taxonomic uncertainties in the genus *Tetropium*. By quantifying and visualizing subtle morphological differences, GM enables more precise species identification, even in cases where traditional morphological approaches fall short. Therefore, providing a robust framework for distinguishing species of quarantine significance, our approach will enhance our understanding of the evolutionary relationships and ecological dynamics within this important genus of longhorn beetles.

## 2. Materials and Methods

### 2.1. Data Acquisition

In order to identify the accuracy of the morphometrics methods, 42 specimens (4 to 5 different males and females for each species) of nine species of the genus *Tetropium* were selected, most of which exhibit cryptic morphologies. The selection criteria included the availability of high-quality images of the pronotum. Images of the thorax for all species were obtained from the internal image database, ImageID, which is utilized by specialists at the United States Department of Agriculture (USDA) Animal and Plant Health Inspection Service (APHIS) Plant Protection and Quarantine (PPQ). When the database did not provide a sufficient number of high-quality images, additional images of accurately identified specimens were sourced from the following external databases: The Old World Cerambycidae Catalog (http://bezbycids.com/byciddb/wdefault.asp, accessed on 1 April 2025), Cerambyx—Cerambycidae of the West Palearctic Region, Neighboring Territories, and Countries of the Former Soviet Union (https://www.cerambyx.uochb.cz, accessed on 1 April 2025), The Atlas of Forest Pests—Europe (https://www.forestpests.eu/pest, accessed on 1 April 2025), and USDA-APHIS-PPQ-IDTools-Cerambycid (https://idtools.org/wbb/cerambycid, accessed on 1 April 2025).

Table 1 presents the species, their natural range (domestic or exotic), and the codes for the specimens that were included in this study. Female specimens were included for each species. Each specimen was selected from a unique geographic location to minimize the incorporation of additional environmental variance. Images were processed using Photoshop version 26.0 (2025, Adobe Creative Cloud) to enhance their quality. Processing involved increasing the image size, cropping to focus on the pronotum, and improving the structural visibility through adjustments to contrast and sharpening.

### 2.2. Morphometrics Analyses

**Figure 1:**
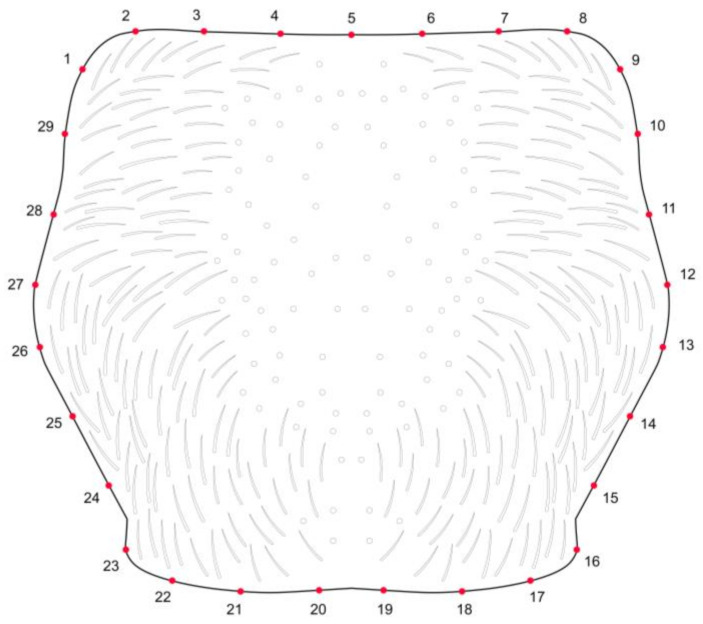
Dorsal view of the pronotum of *Tetropium* species showing the distribution of 29 landmarks along the contour.

Once the images were processed, a total of 29 points were incorporated and resampled as curves. These points were then transformed into landmarks using the function append tps curve to landmarks, representing the entire pronotum contour, using the software TPS Dig2 v2.17 and TPS Util v.1.81 [22] (Figure 1). Procrustes superimposition analysis was applied to the landmarks’ coordinates to remove the effects of size, rotation, and orientation [8]. To determine whether there were higher values of allometry in the data before the analyses, a multivariate regression was performed using the centroid size as the independent variable and shape as the dependent variable.

To visualize the shape variation among *Tetropium* species, the Procrustes-aligned coordinates were organized into a covariance matrix of the individual shape, and a Principal Component Analysis (PCA) was performed as a multivariate ordination method to explore the shape space [23]. The results were plotted, focusing on the two principal components (PCs) that accounted for the most variation. To identify specific shape variations between species, an average shape covariance matrix was generated. The shapes of each species were then exported and superimposed to examine the deviation of landmarks relative to each other, highlighting distinctive geometric features that were useful for taxonomic identification. To determine if there were any statistical differences between species, a Procrustes ANOVA was performed with size and shape as factors. To facilitate the visual examination of shape differences between species, a canonical variate analysis (CVA) was performed. This analysis method is designed to maximize the variation between groups by generating new shape axes. To assess the statistical significance of morphological differences between species, a 10,000-iteration permutation test based on Mahalanobis distances was performed. In order to compare the geometric size of the different species, a centroid size was calculated as a scalar measure representing the square root of the sum of squared distances from each landmark to the configuration’s centroid, and a violin graph was created. All analyses were performed using the R packages geomorph [24] and ggplot2 [25]. The dataset was uploaded as a Supplementary File.

## 3. Results

**Figure 2:**
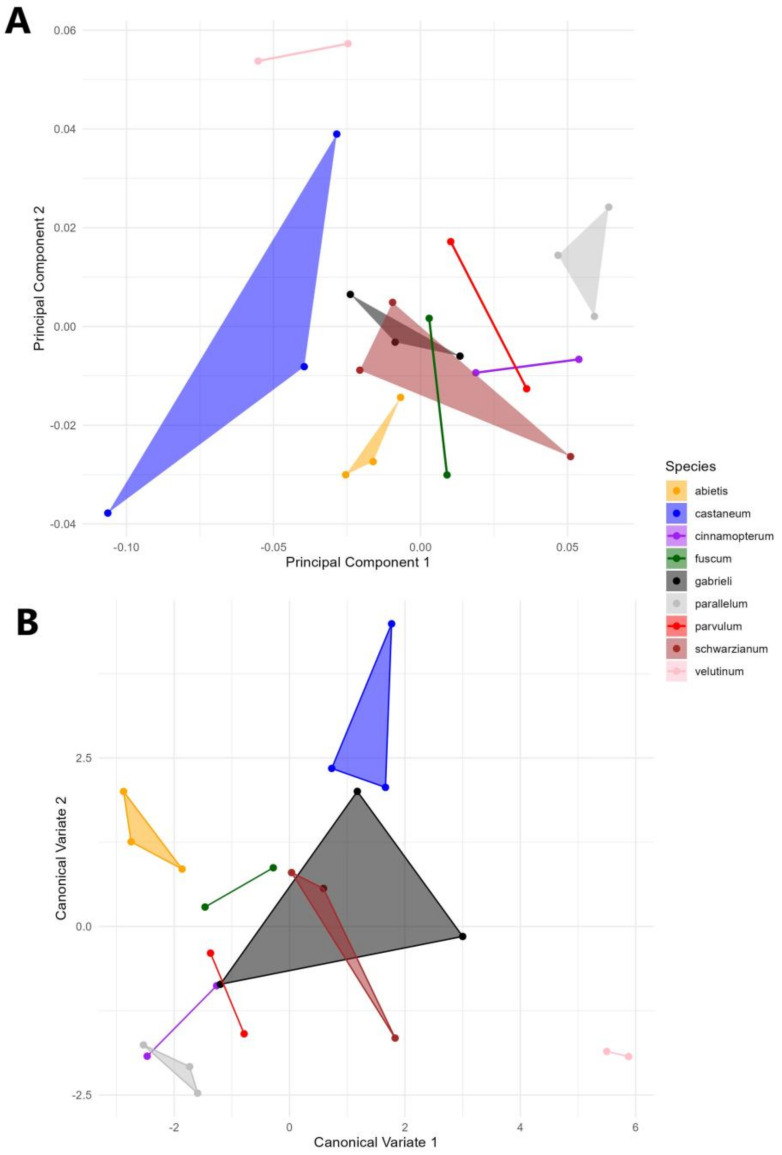
Ordination analysis of the pronotum shape in *Tetropium* species: (**A**) Principal Component Analysis and (**B**) canonical variate analysis.

The multivariate regression analysis showed that the percentage of allometry was only a non-significant 5.5%, indicating that there is no size effect on the data. A Principal Component Analysis (PCA) of the covariance matrix based on individual pronotum shapes showed a particular pattern of variation between species, with clear species differentiation (Figure 2). The first three PCs explained over 65% of the total variation in pronotum shape (PC1 = 40.1%; PC2 = 15.56%; PC3 = 10.4%), providing a robust representation of the overall variation in pronotum morphology. Clear groups of individuals were clustered together, with a principal variation along the PC1, where *T. castaneum* used a big part of the shape space at the right part of the graph. The middle of the graph was mostly occupied by six species, where there was clear overlap between four species, which seemed to have a similar pronotum shape (*T. parvulum*, *T. gabrielli*, *T. cinnamopterum*, *T. schwarzianum*), while on the other hand, two of them (*T. velutinum*, *T. abietis)* showed a clear intraspecies variation, mostly represented by the PC2. On the left of the morphospace, another species, *T. parallelum,* occupied most of the space, with no overlap within the morphospace.

**Figure 3:**
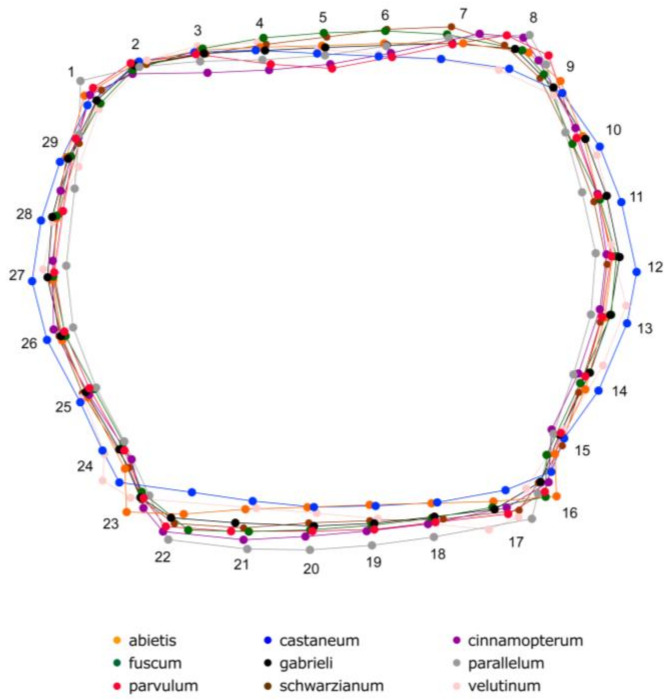
Superposition of the average pronotum shape of *Tetropium* species, with different colors representing each species.

Figure 3 illustrates the average superimposed shapes, showing geometric variation, which can primarily be recognized through the distinct movement of specific landmarks, which contribute to the unique pronotum shapes. The lower corner of the pronotum, defined by landmarks #15, 16, 23, and 24, exhibits pronounced changes with characteristic shapes. For example, *T. castaneum* has a distinctly oval pronotum, with clear elongation of landmarks 12–13 and 26–27, creating a broader shape. In contrast, *T. abietis* shows a distinct repositioning of these landmarks, resulting in a more angular termination at the extremity of the pronotum (#16 and #23). Meanwhile, *T. parallelum* exhibits a more elongated pronotum with an extended caudal section, characterized by significant shifts in landmarks 19–22.

The ANOVA analyses support significant differences in the size of the pronotum shape among the species (F = 2.63; *p* < 0.0001); nevertheless, the centroid size was an evident differentiating factor (F = 0.94; *p* = 0.51). The largest Procrustes and Mahalanobis distances found were between *T. castaneum* and *T. parallelum* (Procrustes: 0.1194; Mahalanobis: 6.225), while the shortest Procrustes distances were between *T. gabrielli* and *T. fuscum* (=0.0394), as were the Mahalanobis distances between *T. gabrielli* and *T. fuscum* (=2.6765) in all four native species. The CVA showed a clear distribution of the shape between the different species regarding their pronotum shape, where the magnification of the shape variation showed that all the species occupied a particular shape, where *T. abietis* was the more distant one with the specimens of *T. schwarzianum*, species which exhibited more variation in the morphospace (Figure 2B).

**Figure 4:**
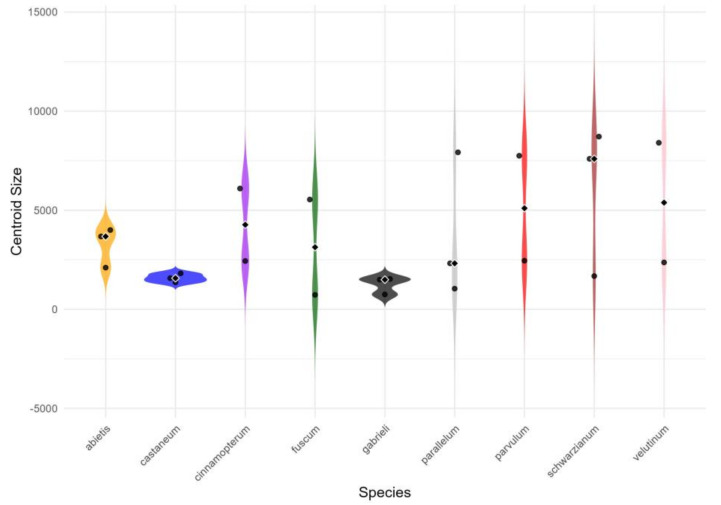
Violin graph of the distribution of the centroid size of the pronotum of *Tetropium* species.

Finally, the centroid size distribution, described in the violin graph (Figure 4), illustrates the variation in pronotum geometric sizes. A clear pattern emerged, showing that *T. castaneum* and *T. gabrieli* exhibited no significant variation in size compared to *T. parallelum*, *T. parvulum*, *T. schwarzianum*, and *T. velutium*.

## 4. Discussion

This study demonstrates the power of geometric morphometric methods for addressing morphological challenges within taxonomically complex groups, such as the genus *Tetropium*. As demonstrated in the results, the shape of the pronotum effectively distinguished the nine analyzed species of *Tetropium*. However, some overlap in pronotum shape was observed in the morphospace among certain native species (see Figure 2). These overlaps were partial and occurred between *T. parvulum* and *T. parallelum*, as well as between *T. parvulum* and *T. cinnamopterum*, despite morphological differences in their shapes. This overlap may reflect evolutionary constraints or ecological adaptations within a shared habitat, suggesting a degree of morphological convergence. The findings highlight the potential of the pronotum shape as a reliable tool for species identification, particularly for distinguishing native species from non-native species. Non-native species, which are serious pests of coniferous trees in North America, are frequently intercepted at ports of entry, often associated with wood pallets, other wood products, or occasionally as hitchhikers in shipping containers.

Gojković et al. [26] demonstrated the utility of GM for resolving cryptic morphological differences and population-level distinctions in beetles (e.g., *Morimus asper* (Sulzer, 1776) complexes on the Balkan Peninsula), where they showed a discordance between delimitation based on traditional morphological traits and the one analyzed with novel genetic and geometric morphometric data. An important contribution to taxonomic identification may be having a database of landmark coordinates for the pronotum of a diverse array of individuals of each species, which would help port specialists and entomologists at entry sites to identify samples in domestic surveys or at ports of entry and to compare the landmarks of an intercepted specimen associated with cargo and achieve a fast and reliable tentative identification, in particular for species of the genus *Tetropium,* which are morphologically similar and difficult to identify by non-specialists in the group. This will ultimately facilitate quarantine decisions and trade. This work represents the first step to the goal of a comprehensive database of landmark coordinates that should include a more considerable diversity of locations, sexual variation, and diversity of different populations associated with different habitats or different hosts, since the diversity/representation of the diversity in this work is limited to some specimens and a few species (there are more than 22 species in the genus *Tetropium*) with high quality, and to specimens that are well positioned in the available images (mesonotum perpendicular to the pin or flat on top of a leveled surface). Furthermore, there is always the possibility of exploring other structures that can yield information in terms of shape variation, the shape of the forewings (elytra), and the shape of the hind wings, which have been shown to be helpful in the separation of morphologically uniform species and genera of seed beetles [27]. Taxonomic identification in insects may reflect an important topic for different areas where misidentification could generate significant problems. This is particularly vital in medical entomology, where the incorrect identification of vectors, such as mosquitoes or triatomines, could compromise disease control strategies and epidemiological surveillance. Dujardin [28] highlighted that morphometric techniques, especially geometric morphometrics, are essential in distinguishing cryptic species and challenges, facilitating accurate diagnostics and supporting efforts to manage and mitigate insect-borne diseases. On the other hand, another example is the study by Jaramillo-O [29], which used applied geometric morphometrics to analyze the wing venation geometry of 11 species of *Anopheles* Meigen 1818 mosquitoes within the subgenus *Nyssorhynchus*, a group containing key malaria vectors in Colombia, supporting entomological surveillance and vector control programs. In agronomic approaches, Lemic et al. [30] emphasized that integrating various monitoring techniques, such as genetic, morphometric, and physiological methods, can significantly enhance the accuracy of pest species identification. This comprehensive approach is essential for developing more effective integrated pest management (IPM) programs, particularly in tackling invasive species like the western corn rootworm, where traditional methods alone may fall short. Finally, the application of GM in this study demonstrates its utility for practical applications, such as quarantine and pest management, by enabling rapid and accurate identification of intercepted specimens. This approach could provide a robust framework for addressing future taxonomic challenges and managing invasive species.

## Figures and Tables

**Table 1:** A list of the species and specimens of *Tetropium* that were used for the study of the differences in shape in the pronotum, along with specimen identification (image code and ID for landmark coordinates).

Species	Origin	Specimen ID
*T. abietis*	Native	146896-Tabietis
		146898-Tabietis
		146899-Tabietis
*T. castaneum*	Exotic	003199-Tcastaneum
		013872-Tcastaneum
		198017-Tcastaneum
*T. cinnamopterum*	Native	010876-Tcinnamopterum
		010877-Tcinnamopterum
*T. fuscum*	Exotic	010880-Tfuscum
		016942-Tfuscum
*T. gabrieli*	Exotic	0001CC-Tgabrieli
		0001CZ-Tgabrieli
		0002CC-Tgabrieli
		040811-Tgabrieli-WK
		309258-Tgabrieli- BIO
*T. parallelum*	Native	002INA-Tparallelum
		146942-Tparallelum
		146943-Tparallelum
*T. parvulum*	Native	146955-Tparvulum
		146956-Tparvulum
*T. schwarzianum*	Native	146966-Tschwarzianum
		146973-Tschwarzianum
		146974-Tschwarzianum
*T. velutinum*	Native	146979-Tvelutinum
		146980-Tvelutinum

## Data Availability

Data will be made available by request to the corresponding author. The data presented in this study are available on request from the corresponding authors due to privacy.

## Supplementary Materials

The following supporting information can be downloaded at: https://www.mdpi.com/article/10.3390/insects16040386/s1. Supplementary File.
